# Antigen Presentation, Autoantigens, and Immune Regulation in Multiple Sclerosis and Other Autoimmune Diseases

**DOI:** 10.3389/fimmu.2015.00322

**Published:** 2015-06-17

**Authors:** Christine Riedhammer, Robert Weissert

**Affiliations:** ^1^Neuroimmunology, Department of Neurology, University of Regensburg, Regensburg, Germany

**Keywords:** autoimmune disease, multiple sclerosis, antigen presentation, autoantigen, T cell, B cell, HLA, MHC

## Abstract

Antigen presentation is in the center of the immune system, both in host defense against pathogens, but also when the system is unbalanced and autoimmune diseases like multiple sclerosis (MS) develop. It is not just by chance that a major histocompatibility complex gene is the major genetic susceptibility locus in MS; a feature that MS shares with other autoimmune diseases. The exact etiology of the disease, however, has not been fully understood yet. T cells are regarded as the major players in the disease, but most probably a complex interplay of altered central and peripheral tolerance mechanisms, T-cell and B-cell functions, characteristics of putative autoantigens, and a possible interference of environmental factors like microorganisms are at work. In this review, new data on all these different aspects of antigen presentation and their role in MS will be discussed, probable autoantigens will be summarized, and comparisons to other autoimmune diseases will be drawn.

## Introduction

Multiple sclerosis (MS) is an inflammatory disease of the central nervous system (CNS), causing a loss of myelin sheaths and a degeneration of axons preferably in the white matter, but also in the cortex (1).

Despite immense exploratory efforts, the pathogenesis and target antigen(s) of MS have so far not or only partially been identified. This problem is also present in other autoimmune disease like rheumatoid arthritis (RA) or type 1 diabetes mellitus (T1D). On the other hand, research has revealed the targets of other diseases like myasthenia gravis (MG) or neuromyelitis optica (NMO), but their exact pathogeneses by the interplay of different immune cells still remain unclear.

Multiple sclerosis is widely thought to be induced and perpetuated by the immune system. T cells are believed to have a central role in the pathogenesis of the disease. It is assumed that defects of central and peripheral tolerance permit an existence of self-reactive T cells, which are activated by antigen-presenting cells (APCs).

As different autoimmune diseases share the feature that risk is conferred by genes that are encoded within the major histocompatibility complex (MHC) locus, antigen presentation generally seems to be of great importance in autoimmune diseases. This review will point out important mechanisms of antigen presentation, autoantigens as well as the contribution of different types of immune cells in autoimmune diseases. The focus will be on MS, but other autoimmune diseases like RA, T1D, MG, NMO, and autoimmune encephalitides (AE) will provide comparisons concerning the genetic background, the ambiguity of the identified or suspected target antigens, and the underlying pathogenic mechanisms.

## Antigen Processing

Peptide fragments that are presented on MHC class I or MHC class II molecules are first processed within the cell before they reach the cell membrane on the respective MHC molecule (2). The processing machineries for MHC class I and MHC class II molecules differ. Peptides derived from the cytosol and the nucleus are presented on MHC class I molecules. For their processing, the proteasome is of paramount importance. Inhibition of proteasomal function can block antigen presentation (3). Interferon (IFN)-γ can induce the immunoproteasome, which leads to the processing of a different set of peptides compared to the constitutive proteasome (4). Apart from the proteasome, also the endoplasmic reticulum (ER) is important for the presentation of peptides on MHC class I molecules. Specialized aminopeptidases are involved in trimming the peptides to a length of 8–10 amino acids (5, 6). Peptides are subsequently loaded on MHC class I molecules. In this process, tapasin and the transporter associated with antigen processing (TAP) are involved, which are expressed in the ER (7). The binding groove of MHC class I molecules is closed, limiting the length of the presented peptides to 8–10 amino acids, even though examples of longer peptides presented on MHC class I molecules exist (8).

Major histocompatibility complex class II molecules bind antigens that are derived from extracellular proteins. These are taken up by endosomes and subsequently processed. The pH in the endosomes is very important for the control of protease activity (9). The binding grooves of MHC class II molecules in the endosomes are normally covered by the invariant chain (10). A specific part of the invariant chain (the CLIP) covers the groove and can be removed by human leukocyte antigen (HLA)-DM (11). Subsequently, peptides that are present in the endosomes can bind in the groove and be presented to T cells. The binding groove of MHC class II molecules is open and presented peptides are generally 14–18 amino acids in length. Under certain conditions, however, also shorter or longer peptides can be presented. While MHC class I molecules are present on all nucleated cells, MHC class II molecules are only present on specialized APCs like dendritic cells (DCs), B cells, and macrophages. In the CNS, microglia are considered as professional APCs (12). Also, astrocytes can present antigen on MHC class II molecules under certain conditions (13). Since the uptake of antigen differs between different types of professional APCs, also the presented set of peptides can differ. In addition, also the intracellular composition of molecules differs regarding the antigen processing machinery (14).

There is cross-presentation of antigens in the MHC class I and MHC class II pathway (15, 16). Different pathways allow this cross-presentation. Phagosomes that take up antigen from the extracellular space can have ER-associated molecules. Also certain immunopeptidases contribute to cross-presentation (17). Cross-presented antigens from extracellular origin are mainly presented on MHC class I molecules of DCs (18).

In regard to autoimmune diseases, the exact sequence of events leading to the presentation of certain sets of autoantigen-derived peptides on APCs in humans is still incompletely deciphered. Potentially, all molecules involved in antigen processing and binding of autoantigens to MHC molecules might influence the emergence of autoimmune disease. Even though the role of the different steps in antigen processing in autoimmune diseases has not been fully established yet, much will be learned over the next years and be of potential importance for the design of new therapies.

## Tolerance

The emergence of an autoimmune disease apparently requires the existence of self-reactive cells which escape central and peripheral tolerance mechanisms and become activated in some way. Self-reactive T cells have been shown to be present both in persons with and without autoimmune disease (19–26). How self-tolerance is disrupted in autoimmune disease, however, remains to be ascertained. The understanding of how tolerance mechanisms work is also the basis for a comprehension of potential deficiencies.

### Central tolerance

The challenge of intrathymic central tolerance induction includes tolerizing T cells reactive with antigens which are not expressed ubiquitously, but only found in particular organs (tissue-restricted antigens, TRAs). The intrathymic expression of TRAs can induce tolerance and prevent development of autoimmune disease (27). This is achieved by negative selection of T cells in the thymic medulla, where T cells exhibiting a high affinity/avidity interaction between their T-cell receptor (TCR) and MHC–self-peptide complexes presented by medullary APCs become apoptotic. Medullary thymic epithelial cells (mTECs) and DCs cooperate to achieve this (28). The thymic transcription factor autoimmune regulator (AIRE) plays a fundamental role in central tolerance induction, as it controls the transcription of genes coding for TRAs (29), and is predisposed, when not working properly, to favor the development of autoimmune disease. Certain single nucleotide polymorphisms (SNPs) in the AIRE gene have been suggested to play a role in autoimmune diseases like RA or MG (30–32). Interestingly, mTECs make use of macroautophagy for presenting antigens also on MHC class II molecules, which enables them to tolerize CD4^+^ T cells (33). Important candidate autoantigens of MS and other autoimmune diseases seem to be present in the human thymus (34–36). Consequently, a lack of presentation of important self-antigens in the thymus cannot fully account for the existence of autoreactive T cells.

Apart from thymocytes recognizing a peptide that is only rarely presented in the thymus, also thymocytes expressing a TCR whose affinity/avidity for a peptide–MHC complex is too low to eliminate the thymocytes during negative selection are thought to be able to elude central tolerance mechanisms (37).

Post-translational modifications of self-antigens in the periphery can also change the affinity/avidity of the interaction between a TCR and a self-peptide–MHC complex. Thus, the affinity/avidity between a TCR and a self-peptide–MHC complex might be low enough to avoid central tolerance induction, but high enough to lead to an activation of autoreactive T cells in the periphery after a post-translational modification of the antigen. For example, thiopalmitoylation occurs frequently with proteolipid protein (PLP), a putative autoantigen in MS, and it was found that post-translationally modified PLP is more encephalitogenic than the unmodified form (38). Also citrullinated proteins were found more frequently in MS lesions than in control brain tissue (39).

Another interesting aspect is the structural nature of the interaction between a TCR of a potentially self-reactive T cell and its respective peptide–MHC complex in the thymus. An analysis of the crystal structure of a TCR and a self-peptide–MHC complex in an MS patient showed that the TCR bound in a tilted way to the peptide–MHC complex (40). Such structural deviations might result in a low affinity/avidity of the interaction between TCR and self-peptide–MHC complex, so that the T cell can escape from central tolerance. In the periphery, however, the quantity of self-antigen is higher and the affinity/avidity might then be high enough to contribute to the pathogenesis of autoimmune disease. It was shown that autoreactive T-cell clones both in MS and T1D exhibited a lower recruitment of self-peptide–MHC complexes than T cells from the same patients specific for viral peptides. Nevertheless, the autoreactive T-cell clones proliferated vigorously in response to self-antigen, indicating that TCR signaling is still functional in these cells (41). Again, these facts might support an escape of negative selection and a possible activation of these cells in the periphery.

### Peripheral tolerance

When self-reactive T cells escape central tolerance mechanisms, peripheral tolerance mechanisms are required, which include ignorance, i.e., the physical separation of autoreactive cells and their target, the deletion of autoreactive cells, their inhibition, e.g., by a lack of costimulatory factors and their suppression by regulatory T cells (42).

An important factor for the inhibition of T cells is the function of the cytotoxic T-lymphocyte-associated protein 4 (CTLA-4 = CD152). Its role in MS has been investigated in many studies. A recent meta-analysis of genetic polymorphisms of the CTLA-4 gene did not reveal any associations with MS (43). However, there are several studies pointing toward a significance of costimulatory mechanisms for MS. Myelin-reactive T cells from MS patients were observed to be less sensitive to a lack of costimulation than T cells from controls (44–46). Also, another study found increased T cells insensitive to costimulation in MS patients, which predominantly produced Th1 cytokines (47). In active MS, increased CD80^+^ cells were found (48).

An important mechanism for the deletion or “activation-induced cell death” of autoreactive cells is the interaction of Fas and Fas ligand, which can induce apoptosis in autoreactive T cells. A higher resistance of T cells to apoptosis in MS patients has been reported in several studies which found decreased expression of Fas and a lower rate of apoptosis induced by stimulation with mitogen in T cells from MS patients (49, 50). New data indicate that Th1 cells seem to be more susceptible for Fas-mediated apoptosis than Th17 cells, both in MS patients and healthy controls (51).

Regulatory T cells are probably the most important contributors to the maintenance of peripheral tolerance. They are mainly defined by the expression of the transcription factor Foxp3 (52). Foxp3^+^ regulatory T cells are able to suppress proliferation of CD4^+^CD25^−^ T cells (52, 53). It was found that interactions of thymocytes with MHC class II molecules expressed by mTECs were important for the induction of regulatory T cells (54). The possible role of different regulatory cell types in MS is discussed below in the respective sections.

## Immune Pathogenesis

The CNS as the target organ of the autoimmune response in MS is a special one. It is only a short time ago that it was recognized that the CNS is physiologically supervised by immune cells. Thereby, memory T cells are thought to play an important role (55).

**Figure 1:**
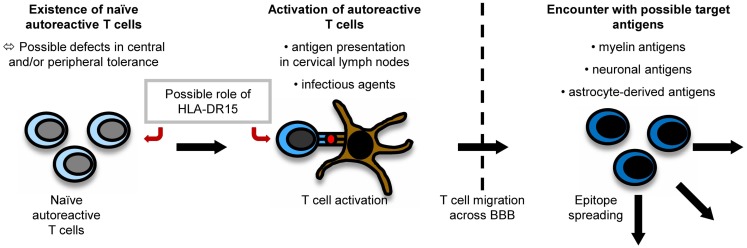
**Pathogenesis of MS**. The exact pathogenesis of MS is not clear yet. However, it can be imagined that naïve autoreactive T cells exist because of imperfections in central and peripheral tolerance mechanisms. They might then become activated by antigen presentation in cervical lymph nodes or effects of molecular mimicry. Antigen presentation on disease-associated HLA-DR15-molecules might influence both the emergence and activation of autoreactive T cells. When autoreactive T cells reach the CNS by crossing the BBB, they encounter their target antigen and start an inflammatory cascade, in which other antigens become unveiled, triggering the process of epitope spreading.

It is believed that myelin-specific T cells have a crucial role in the pathogenesis of MS (56). Naïve myelin-specific T cells, which cannot access areas outside the secondary lymphoid tissues (57), are thought to be activated in the periphery, gain access to the CNS, and then be reactivated by CNS-resident cells presenting self-antigen (37). The CNS is extraordinarily shielded from the periphery by the existence of the brain–blood barrier (BBB) and the brain–cerebrospinal fluid (CSF) barrier. For activated T cells, there are three routes to enter the CNS, i.e., by crossing the BBB or the blood–CSF barrier or by migrating through the carotid arteries into the subarachnoid space (58). The most important pathogenic mechanisms in MS are outlined in Figure 1.

### Peripheral activation of T cells

How T cells specific for CNS antigen are activated in the periphery remains unclear. However, their activation could result from a trigger by an infectious agent or from the presentation of myelin antigens in the cervical lymph nodes (37). Concerning the latter mechanism, CSF is drained via the nasal lymphatic system to the cervical lymph nodes. In experimental autoimmune encephalomyelitis (EAE) induced in marmosets, a relevant animal model of MS, myelin components were detected to be increased in APCs in cervical lymph nodes (59). A study using fine-needle biopsy material of cervical lymph nodes of MS patients and controls also detected more macrophages containing myelin basic protein (MBP) and PLP in MS patients than in controls (60). Therefore, APCs presenting myelin fragments in the cervical lymph nodes might contribute to an activation of naïve self-reactive T cells.

### Role of infectious agents

Apart from this concept, also infectious agents are considered to play an important role in triggering autoimmune diseases. In this context, a cross-talk between the innate and adaptive immune system might be relevant: infectious agents can activate APCs via Toll-like receptors (TLR). APCs can then contribute to the activation of self-reactive T cells (61).

Structural similarities between antigens of infectious agents and myelin proteins (molecular mimicry) can induce an activation of naïve autoreactive T cells which recognize peptides derived from infectious agents and self-antigens. Novel data indicate that cross-reactivities do not necessarily require nearly identical amino acid sequences. They also occur when some important motifs are conserved which make sure the overall structures of TCR–peptide–MHC interaction are similar (62). This suggests that cross-reactivities may happen frequently. There has been intensive research on possible infectious triggers of MS: it has been shown that T-cell clones specific for an immunodominant epitope of MBP, namely MBP_85–99_, also recognize viral peptides, e.g., of Epstein Barr virus (EBV), influenza A virus, herpes simplex virus, human papilloma virus (63), or human herpesvirus-6 (64). Concerning EBV, for example, MS patients seem to exhibit higher antibody titers against certain antigenic components of the virus than controls even before the onset of MS (65). In EAE experiments, it could also be shown that a peptide from *H. influenzae* mimicking a PLP-peptide can actually induce CNS disease (66).

In the context of molecular mimicry, also self-mimicry has been observed. Transgenic myelin oligodendrocyte glycoprotein (MOG)-deficient mice expressing a MOG-specific TCR develop EAE due to a cross-reactivity between a MOG epitope and neurofilament NF-M (67). Such cross-reactivities could play a role in the induction of axonal damage also in human MS.

Independently from cross-reactivities, infectious agents can lead to a disruption of tolerance to self-antigens by bystander activation. For example, demyelination can be induced when certain immunodeficient (RAG2^−/−^ transgenic) mice are infected with mouse hepatitis virus (MHV), even though the CD8^+^ T cells they possess are neither specific for MHV nor for CNS antigen, when their T cells are activated by the antigen they recognize (68).

Recently, besides molecular mimicry and bystander activation, another interesting mechanism has been proposed: myelin-specific CD8^+^ T cells expressing a dual TCR specific for both MBP and viral antigens have been discovered. The activation of such T cells during viral infection might also induce autoimmune reactions (69).

Besides infectious agents, commensal microbiota could be of importance in the pathogenesis of the disease. EAE in mice expressing a transgenic TCR for MOG was found to depend on the presence of the commensal gut flora (70).

### Epitope spreading

During the course of an autoimmune disease, otherwise physiological immunological mechanisms like epitope spreading set in, which contribute to the perpetuation and diversification of the ongoing immune response. Epitope spreading means the expansion of the immune response to epitopes that are different from the initially targeted ones. This process is physiological and helpful in the fight against pathogens, but it also seems to play an important role in the emergence of autoimmune responses.

In EAE, it could be shown that the immune response is first focused on a certain epitope and then spreads to other epitopes during the chronification of the disease (71, 72). Apart from intramolecular epitope spreading (e.g., within different MBP epitopes), also intermolecular epitope spreading, e.g., from MOG to MBP, has been observed in different EAE models (71, 73, 74). In different animal models of MS, it could also be shown that epitope spreading can begin in the CNS (75). Interestingly, also in an animal model using the CNS-resident virus Theiler’s murine encephalomyelitis virus for disease induction, T-cell reactivities against certain myelin epitopes emerged during the course of the disease, which were not due to molecular mimicry (76). Epitope spreading was reported to be associated with clinical relapses in animal models, as T cells reactive with epitopes the immune response had spread to could induce disease in other animals (74).

Both intramolecular (24, 25, 77–79) and intermolecular (80) epitope spreading has been observed in MS patients as well. However, it remains to be proven that this process also plays a pathogenic role in the disease, as some studies could not detect any associations with clinical exacerbations (77, 78).

Epitope spreading is also involved in other autoimmune diseases, complicating the search for the initial target antigens of the autoimmune response and complicating also the development of potent therapies which should ideally operate in all or many patients. Further understanding of this process will be crucial for designing efficient therapies.

## Immune Cells Involved in the Pathogenesis of MS

### Role of CD4^+^ T cells

CD4^+^ T cells are widely considered major players in the pathogenesis of MS.

**Figure 2:**
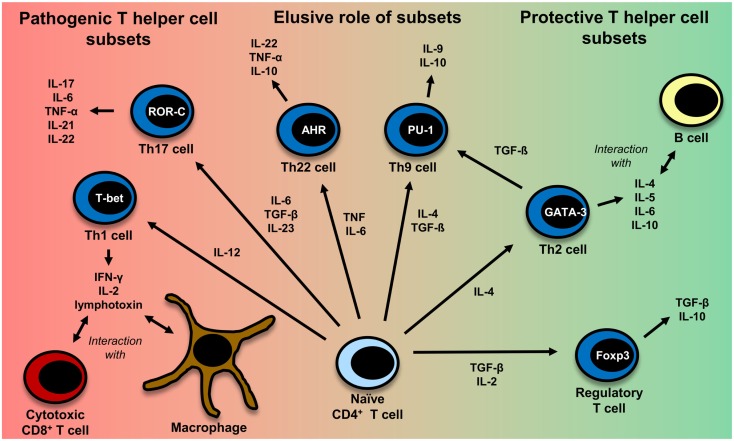
**CD4^+^ T-cell differentiation**. Naïve CD4^+^ T cells can differentiate into different T helper cell subsets. In MS, Th1 and Th17 cells are regarded to be disease-promoting, whereas regulatory T cells and Th2 cells seem to exert a protective effect. The exact role of Th9 and Th22 cells remains to be further clarified. Th1 cells, which predominantly produce TNF (95), IFN-γ, IL-2 (332), and lymphotoxin-α (333), are induced by IL-12 (95). Their master regulator is the transcription factor (TF) T-bet (334). They mainly interact with cytotoxic CD8^+^ T cells and macrophages (335). Th2 cells, which mainly interact with B cells (335), are induced by IL-4 and secrete IL-4, IL-5, IL-6, and IL-10 (101, 333, 335). GATA-3 is the most important TF of Th2 cells (336). IL-6, TGF-β (93), and IL-23 (95) are important cytokines for the induction of Th17 cells producing IL-17, IL-6, TNFα (95), IL-21 (94), IL-22 (337). RORγt is the master regulator of this T-cell subset (338). Interestingly, TGF-β is also necessary for the induction of regulatory T cells, whereas IL-6 must be absent in this case (93, 117). The master regulator for the induction of regulatory T cells is the TF Foxp3 (52, 339). Regulatory T cells then produce TGF-β and IL-10 (117). Recently also Th9 cells and Th22 have been identified to be a discrete T-cell subset. The induction of Th9 cells is promoted by IL-4 and TGF-β (340). Th9 cells produce IL-9 and to a lesser extent IL-10 (340, 341). Under the influence of TGF-β, Th2 cells can switch their phenotype and become Th9 cells (342). Master regulators for Th9 and Th22 cells have not been identified so far. Important factors for the induction of Th9 cells are PU.1, IRF, BATF, different STAT factors, and TGF-β-induced SMADS (341, 343). Priming of IL-22 producing Th22 cells is promoted by IL-6 and TNF (103). The transcription factor aryl hydrocarbon regulator (AHR) seems to exert an important influence on IL-22 production (344).

This is in part due to the fact that most of the genetic susceptibility for MS is associated with certain MHC class II alleles (81). CD4^+^ T cells have also been detected in MS lesions (82). Evidence also comes from a humanized mouse model: transgenic mice expressing the MS-associated DR2-molecule (DRA*0101/DRB1*1501), an MBP-specific TCR derived from MS patients and human CD4 develop disease with symptoms very similar to those in MS and more severe symptoms than mice lacking CD4 expression (83). It is not clear yet, which CD4^+^ T helper cell subset (Th cell) exerts the most important influence on the disease. The subsets which are discussed most are Th1 cells and Th17 cells. Figure 2 gives an overview of different T-cell subsets, their signature cytokines, and important transcription factors.

#### Th1 Cells

Th1 cells have been and are seen as causative agents in MS, since MS-resembling animal models are driven by this T helper cell subset. There is also evidence that this T-cell subset might play a major role in human MS.

Administration of an altered peptide ligand (APL) based on an immunodominant epitope of MBP led to cross-recognition of MBP and the APL, resulting in exacerbations in some patients. The phenotype of the reactive T cells was skewed toward Th1 (84). Also other studies showed (partly by determination of chemokine receptor expression) that Th1 cells are especially increased in MS patients suffering a relapse compared to healthy controls or patients in remission (85–87). In PLP-specific T-cell lines isolated from MS patients during acute attacks, Th1 cytokines were found to be dominant (88) and also MBP-specific T-cell lines in MS patients produced significantly more IFN-γ than T-cell lines from controls (89). A correlation between IFN-γ-secretion (after stimulation with phytohemagglutinin) and contrast-enhancing lesions in MR was also detected (90). Higher levels of myelin-specific high-avidity CD4^+^ T cells were found in MS patients than in healthy controls, which exhibited a Th1 phenotype and higher IFN-γ production in MS patients (91). Recently, EAE experiments showed that Th1 cells first infiltrate the CNS and are suppressed by regulatory T cells, which stops progression of the disease, whereas Th17 cells infiltrate the CNS later during the disease course (92).

#### Th17 Cells

In the last years, interleukin (IL)-17 producing Th17 cells have moved to the focus of research. Important factors for the induction, differentiation, and maintenance of the Th17 cell repertoire are IL-6, transforming growth factor (TGF)-β (93), IL-21 (94), and IL-23 (95). IL-23-deficient mice have been shown to be unable to develop EAE (95). Interestingly, the development of regulatory T cells also requires TGF-β in the absence of IL-6 (93). IL-17 transcripts have been shown increased especially in chronic silent MS lesions (82) and IL-17 positive cells were also found increased in MS lesions (96). In MS patients, more Th17 cells have been detected than in healthy controls and NMO patients (97). A study investigated T-cell subsets in patients with an aggressive disease course who received high-intensity chemotherapy followed by autologous stem cell transplantation, which induced remission in all patients. Interestingly, Th17 cell numbers were still decreased 1 year after transplantation, whereas Th1 cells had reached normal levels, which might speak for an association between the decreased Th17 cells and the remission (98). Higher IL-17 production by myelin-reactive T cells has been reported in MS patients than in controls (99).

#### Th9 Cells and Th22 Cells

Recently, novel T-cell subsets have been detected whose role in MS has already begun to be investigated: Th9 cells reside mainly in the skin, where IL-9 might also play a role in the production of other cytokines like IFN-γ, IL-13, or IL-17 by T cells in the skin (100). Besides Th1 and Th17 cells, also Th9 cells could be shown to induce a milder form of EAE (101).

Similar to Th9 cells, Th22 cells were identified as a distinct T-cell subset to be present in the skin a few years ago (102, 103). Increased fractions of (myelin-reactive) Th22 cells have been reported in MS patients (104).

#### Regulatory T Cells

In MS patients, there does not seem to be a reduction of regulatory T cells in number (105–110), but there is some evidence that regulatory T cells might suppress autoreactive T cells less efficiently compared with healthy controls: regulatory T cells from MS patients seem to be less able to suppress effector T cells (106–108, 110–112) and have a lower cloning frequency than regulatory T cells from healthy controls (106). However, it must be stated that some of these studies assumed the CD4^+^CD25^hi^ phenotype as characteristic for regulatory T cells. However, after Foxp3 had been discovered, it became clear that by far not all regulatory T cells express CD25 at high levels (53). New regulatory T cells either mature in the thymus or stem from an expansion of regulatory T cells in the periphery. The latter mechanism physiologically gains importance with increasing age. In MS patients, there seems to be a selective lack of regulatory T cells released from the thymus, to which the observed functional impairment might be ascribed. The difference in thymic release of regulatory T cells between MS patients and healthy controls vanishes with increasing age of patients and controls (113–115). This might also partly explain why a functional impairment of regulatory T cells was detected in (younger) MS patients with relapsing-remitting disease (RR-MS) but not in (older) patients with secondary-progressive MS (SP-MS) (109). Interestingly, Th17 cells can only be suppressed by a special subset of regulatory T cells expressing CD39, which were found reduced in number and less functional in RR-MS patients (but not in SP-MS patients) (116).

In EAE experiments, different observations concerning the function of regulatory cells during relapses were made: a study observed that regulatory T cells succeeded in suppressing naïve splenic autoreactive T cells, but not effector T cells from the CNS (in a cytokine milieu containing IL-6 and TNF) at the peak of disease (117). In another EAE study, however, regulatory T cells were found fully functional also during the peak of disease and were shown to alleviate disease by a putative suppression of Th1 cells (92). Similarly, human T cells showed an enhanced expression of genes responsible for suppression of IFN-γ (118). These findings might indicate that during acute attacks the immune system starts a counterregulation, which might lead to remission. Interestingly, regulatory T cells were found increased in the CSF of MS patients compared to controls regardless of an active or inactive disease state (108). In a study investigating autoimmune responses after bone marrow transplantation in rats at the peak of EAE, an induction of regulatory T cells was shown, which are probable to make an important contribution to the achieved remission observed (119).

### Role of CD8^+^ T cells

Recently, also CD8^+^ T cells have drawn more attention. They are more frequently encountered in MS lesions than CD4^+^ T cells (120). CD8^+^ T-cell clones detected in brain lesions were shown to be still present in blood and CSF after several years (121). Another fact supporting the pathogenic role of CD8^+^ T cells is that MHC class II molecules interacting with CD4^+^ T cells are mainly expressed on professional APCs. However, MHC class I molecules are expressed on all nucleated cells, including oligodendrocytes or astrocytes and CD8^+^ T cells can interact with peptides bound to the MHC-I complex. In this context, CD8^+^ T cells were shown to be able to cause axonal damage (122). Increased reactivities of MBP-specific CD8^+^ T cells were shown to be present in MS patients compared with healthy controls. The CD8^+^ T cells identified by this study were predominantly memory T cells (CD45RO^+^) (123). An increased proportion of memory CD8^+^ T cells was also reported in the CSF of MS patients (124, 125). Elevated levels of granzyme, which is released by cytotoxic T cells, were found in the CSF of patients during relapse (126). So far, many attempts have failed to demonstrate stable disease-relevant CD8^+^ T-cell expansions to specific CNS-derived antigens in blood from MS patients. This is in contrast to some interesting studies in EAE, in which the relevance of myelin-specific CD8^+^ T cells has been shown. Importantly, CD8^+^ T cells specific for MBP can mediate a form of EAE in which a predominance of lesions is seen in the brain rather than in the spinal cord and less inflammation can be observed than in classical EAE (127).

On the other hand, also regulatory autoantigen-specific CD8^+^ T-cell populations exist, which are able to destroy pathogenic CD4^+^ T cells or mediate a suppression of their proliferation by interacting with DCs. Interestingly, these suppressive capabilities were found to be reduced during relapses (128–130).

### Role of B cells

**Figure 3:**
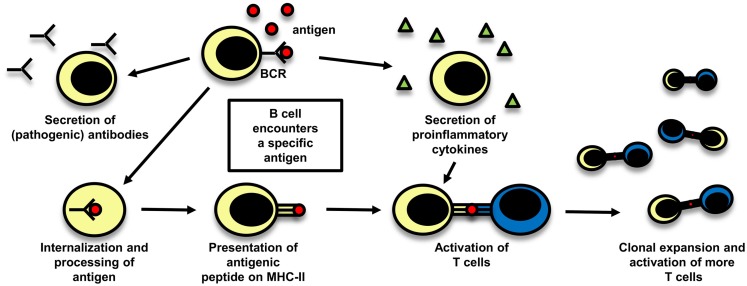
**The role of B cells in MS**. B cells can play a role in autoimmune disease by different mechanisms which are depicted here: they can secrete potentially pathogenic antibodies, but also their function in cellular immune mechanisms are of importance, either by secreting proinflammatory cytokines and thereby influencing other immune cells or by acting as antigen-presenting cells. After B cells have encountered their specific antigen, they process it, present it on MHC and can become activated. As they are able to expand clonally, they are then capable to activate a big number of T cells.

Even though T cells are widely believed to play the central role in the pathogenesis of MS, there is also evidence supporting a pathogenic role of B cells. The importance of a possible role of B cells in MS is reflected in a study with rituximab, a B-cell depleting anti-CD20 monoclonal antibody (mAb), which was effective in reducing both lesion load measured by MRI and clinical relapses (131). The role of B cells in MS is schematically depicted in Figure 3.

Most MS patients show IgG oligoclonal bands in their CSF, indicating a local antibody production in the CNS (132). However, the targets of these locally produced antibodies have not yet been fully revealed. MOG-specific autoantibodies have been discovered in acute lesions of MS patients (133, 134).

Apart from their ability to secrete antibodies, the cytokines secreted by B cells can also influence T-cell proliferation in MS patients (135). A further important aspect in their role in cellular immunity is that they can serve as APCs (136). Their ability to expand clonally allows them to activate many T cells. In an EAE model depending on B and T cells, the functions of B cells as APCs were shown to be necessary for the induction of EAE instead of their ability to secrete antibodies (137). Memory B cells from RR-MS patients were shown to induce an increased response of CD4^+^ T cells to myelin antigens compared to memory B cells from healthy controls, also pointing toward a role of B cells as APCs (138). Clonally expanded B cells have been detected even in the CNS of recently diagnosed MS patients (139). Another interesting aspect concerning B cells as professional APCs is that the B-cell receptors (BCR) consisting of membrane-bound antibodies mostly recognize conformational epitopes of a protein, whereas T cells recognize linear peptides, processed before by APCs (140). The epitopes of an antigen presented by B cells might therefore be different from those presented by thymic epithelial and DCs, including the possibility that T cells specific for epitopes presented by B cells might escape central tolerance induction more easily.

Defects in peripheral (but not central) B-cell tolerance have been reported in MS patients, whereas, interestingly, defects in both peripheral and central B-cell tolerance have been detected in RA and T1D patients (141).

There are also B cells which can adopt regulatory functions, e.g., IL-10-secreting B cells, which can inhibit cytokine secretion (142). The induction of these IL-10-secreting B cells has been found to depend on the presence of IL-21 (which can be secreted, e.g., by Th17 cells) and CD40-dependent cognate interactions with T cells, whereas it can be inhibited by the presence of IFN-γ and TGF-β (143). It is not fully clear yet if the regulatory function of B cells might be defective in MS. Data on a possible reduction of IL-10-secreting B cells in MS patients are contradictory, which is probably also due to different study designs (144–146). Interestingly, parasite infection with helminths in MS patients can induce these IL-10-secreting regulatory B cells (147).

### Role of other professional APCs

Apart from B cells, also other types of APCs have been shown to be of importance for antigen presentation in MS. Most work regarding the role of APCs in inflammation of the CNS has been conducted in rodent models. Unfortunately, the knowledge about APCs in human MS is still incomplete and not all gathered knowledge can be translated from rodents to humans. DCs are very important for linking innate and adaptive immune responses. Different types of DCs have been described in humans, namely the blood DCs plasmacytoid (p)DC (BDCA2), CD141^+^ DC (BDCA3), and CD1c^+^ (BDCA1), as well as the skin DCs, the Langerhans cells, and CD14^+^ DCs and inflammatory DCs in non-lymphoid tissue (148). All types are capable of presenting antigen, but differ in their expression pattern of molecules, migratory activity, cytokine secretion, and capacity for antigen presentation (149). For example, human CD141^+^ DC (BDCA3) express mainly TLR3, human CD1c + DC (BDCA1) mainly TLR2 and pDC (BDCA2) mainly TLR7 and TLR9 (150). This leads to different capabilities to react to certain pathogens in the environment. TLR stimulation itself results in DC maturation (151). DCs are required for the priming of naïve T cells (152). Also the capability to cross-present antigen differs between different types of DCs (153). Even though DCs are obviously very important for the immunopathogenesis of MS, so far only limited data are available (154). Also macrophages have been shown to be capable to present antigen in MS (155). In rodent models, perivascular phagocytes in the CNS which present CNS antigens to T cells are of paramount importance in lesion development (156). Such cells can also be found in human MS (157). Within the CNS, microglia can serve as professional APCs (158). A change in the activation pattern of microglia is already seen very early in MS, which could potentially also speak for an increased capacity of antigen presentation (159, 160). Also astrocytes can express MHC class II molecules under certain conditions and present antigens in rodents (13, 161). In humans, so far the role of astrocytes in antigen presentation in CNS inflammation is still incompletely defined.

## Genetics of Multiple Sclerosis

Multiple sclerosis is not a classical hereditary disease. It is rather defined as a complex genetic disease. This is supported by twin studies, in which for monozygous twins the concordance rate is around 30%, and by findings in siblings of affected individuals who carry a risk of about 2% (162). Most probably, a combination of genetic and also environmental risk factors like smoking (163) contributes to its pathogenesis.

More than 100 risk loci have been identified so far in genome-wide association studies (164), among which the variations within the HLA complex play the most important role. The most significant gene codes for the MHC class II, DR (81). The haplotype HLA-DR2 confers the largest part of the genetic risk for MS in Caucasians. In this MS-associated haplotype, two HLA-DRβ-chains (HLA-DRB1*1501 and HLA-DRB5*0101) pair with the α-chain HLA-DRA*0101, resulting in two heterodimers DR2a (HLA-DRA*0101, HLA-DRB5*0101) and DR2b (HLA-DRA*0101, HLA-DRB1*1501) expressed on the surface of APCs. In this haplotype, three alleles are in linkage disequilibrium and therefore mostly inherited together: HLA-DRB1*1501, HLA-DRB5*0101, and HLA-DQB1*0602 (165). Among these three alleles, HLA-DRB1*1501 confers the greatest genetic risk for MS. Other alleles with increased risk are HLA-DRB1*0301, HLA-DQB1*0201, HLA-DRB1*1303 (81). An association of the HLA-DR15 haplotype in MS with younger age at disease onset was reported, but no correlations with the clinical course, prognosis, or disease type could be detected (166). In contrast, some HLA-A alleles can be attributed some protective effect, most notably the allele HLA-A*0201, but also HLA-A*6801, HLA-A*0205, and HLA-A*0206 have recently been identified as protective (81). The expression of HLA-A*0201 in a humanized transgenic mouse model also protected animals from developing disease, possibly by influencing negative selection processes in the thymus (167).

Apart from the variations in the MHC-locus, 110 other risk-conferring genes have been identified up to now in genome-wide association studies (164). Interestingly, a magnitude of them are genes related to the immune system, which corroborates the hypothesis that MS is an autoimmune disease. Only some important genes can be discussed here. Genes regulating IL-2 signaling, which plays a pivotal role in T-cell activation, have been proposed to be associated with MS, e.g., the IFL-3 gene which regulates IL-2 expression by T cells, but also associations with the IL-2 receptor have been described (81, 164). Concerning the cytokine pathway, also genes coding for IL-7 and its receptor have been linked with a higher risk for MS. Less suppressive effects of regulatory T cells in MS patients was observed in the presence of cells expressing IL-7Rα (110), providing a possible link between the associated gene and a possible function in the disease. Also associations with genes coding for molecules involved in the costimulatory pathway like CD40 or CD80 have been detected. The possible role of defects in this mechanism has been discussed above. Several genes coding for signal transduction molecules (like STAT-3) have been described as well, which are highly relevant for immune cell function. There are also genes related to environmental risk factors such as vitamin D (e.g., CYP27B1) (81).

Table 1 shows some of the susceptibility loci of MS, RA, T1D, and MG in comparison. Even though this list is by far not exhaustive, some interesting aspects can be comprehended: associations with MHC molecules are found not only in MS, but also in RA, T1D, MG, and other autoimmune diseases (168–171). There are also genes coding for molecules involved in similar costimulatory pathways like CD86 or CTLA-4, which contribute to the strength of the peptide–MHC interaction, or for cytokine pathways like IL2RA or TNF in the respective diseases (81, 168). This shows that there may be similar genetic traits leading to the development of different autoimmune diseases.

**Table 1:** **Susceptibility genes in different autoimmune diseases**.

Risk locus	Function of associated gene/remarks	Reference
**Multiple sclerosis**		
HLA-DRB1*1501	Antigen presentation	(81)
HLA-DRB1*0301		
HLA-DQB1*0201		
IL2RA = CD25	Mediation of IL-2 stimulation of T cells	(81)
CD86	Role in costimulation, expressed on APCs	(81)
TNFRSF1A	Implication in TNF pathway	(81)
TNFRSF14		
TNFSF14		
CYP27B1	Activation of vitamin D precursor	(81)
**Rheumatoid arthritis**		
HLA-DRB1*0401	Antigen presentation	(170, 171, 297–299)
HLA-DRB1*0404		
HLA-DRB1*0101		
PTPN22	Down-regulation of T-cell activation	(168, 300)
IL2RA = CD25	Mediation of IL-2 stimulation of T cells	(168)
CTLA-4	Binds CD80 on APCs, inhibits T-cell activation	(168)
TNFAIP2	Implication in TNF pathway	(168)
**Type 1 diabetes**		
HLA-DQB1*0302	Antigen presentation	(171, 301, 302)
HLA-DQ2		
HLA-DRB1*0301		
HLA-DRB1*0401		
HLA-DRB1*0404		
PTPN22	Down-regulation of T-cell activation	(168, 303)
IL2RA = CD25	Mediation of IL-2 stimulation of T cells	(168, 304)
CTLA-4	Binds CD80 on APCs, inhibits T-cell activation	(168, 305)
interferon-induced helicase 1 (IFIH1)	Pathogen recognition receptor for viral infection	(168, 306, 307)
INS	Codes for insulin	(308)
**Myasthenia gravis**		
HLA-B*08	Antigen presentation, association found in early-onset MG	(169)
PTPN22	Down-regulation of T-cell activation, association found in early-onset MG	(169)
TNIP1 (= TNFAIP3-interacting protein)	Reduction of NFκB1 activation, association found in early-onset MG	(169)
**Neuromyelitis optica**		
HLA-DPB1*0501	Antigen presentation, association in Asian, but not Caucasian NMO patients	(309, 310)
**Autoimmune encephalitis**		
None reported so far		

*Some of the major disease susceptibility genes in MS, RA, T1D, MG, NMO, and AE reveal interesting similarities*.

Genetic studies are able to reveal potential associations of certain genes with autoimmune diseases, but subsequent functional studies are necessary to determine the functional relevance of the identified genes. The functional relevance of most of the genes associated with different autoimmune diseases still remains to be clarified.

The relatively strong association of genes coding for MHC molecules with different autoimmune diseases suggests that the respective genetic variants might affect antigen presentation in a way that the emergence of an autoimmune disease is facilitated. Structural analyses of the MHC loci in different autoimmune diseases revealed certain characteristics of the binding groove, potentially leading to a preferential presentation of certain peptides (171). In general, MHC class II molecules are involved both in negative selection in the thymus by presentation of self-peptides and in antigen presentation in the target tissue. However, the exact mechanisms how risk is conferred by the MHC molecules still remain sketchy. An interesting new aspect was recently observed: in HLA-DR15^+^ individuals, increased autologous T-cell proliferation was found. In addition, the authors discovered that the HLA-DR15 risk allele in MS is presented on itself, suggesting a possible role of this haplotype as an autoantigen and as a contributor to autologous proliferation (172). Interestingly, also in RA, DRB1*0401-β-chain-peptides were found to be presented by themselves (173).

A future further understanding of the risk-conferring mechanisms of the HLA genes will provide important new insights into the pathogenesis of the respective autoimmune diseases.

## Discussed Autoantigens in MS

Principally, possible candidate autoantigens in MS include myelin antigens, neuronal antigens, and astrocyte-derived antigens. So far, most research has been done on myelin proteins, but there is evidence that also other antigens could serve as possible autoantigens. In the following, some of the antigens derived from the myelin sheath are discussed in detail, which drew most attention of research in the past. Even though these are the most promising candidate autoantigens in MS, it will become clear that the data are not always consistent.

### Myelin basic protein

Myelin basic protein is the second most abundant myelin protein after PLP. It is the only myelin component that can be found in both central and peripheral myelin. Thus, a peripheral activation of MBP-specific T cells seems more viable than of T cells reactive with other myelin components, from which they are normally separated.

It has been shown that there are MBP-reactive T cells in MS patients and healthy controls (19, 20). Several studies found significantly more IFN-γ-secreting T cells in response to different MBP–peptides in MS patients than in controls, suggesting a quantitative increase of T cells exhibiting an activated phenotype (89, 174–176). Significantly more MBP-specific T-cell lines were found in the CSF of MS patients than of controls (23).

The central region of MBP_84–102_/MBP_83–99_, which can bind to several HLA-DRB molecules, was identified as immunodominant in several studies (177, 178). However, results varied with respect to differences in reactivities to MBP_84–102_ between MS patients and healthy controls. While some studies found increased reactivity to MBP_84–102_ in MS patients (20) or an enhanced response to MBP_83–99_ in MS patients during relapse (87), others could not discover any discrepancies between the two cohorts (179–181). Krogsgaard et al. showed by staining of CNS tissue of HLA-DR2-positive patients with a mAb specific for the HLA-DR2:MBP_83–99_ complex that this MBP epitope is locally presented in the CNS of MS patients, supporting a possible role as an autoantigen (182). Other epitopes described as immunodominant are MBP_13–32_ (177) and MBP_144–163_ (177)/MBP_143–168_ (23)/MBP_151–170_ (87). Interestingly, for high-affinity T cells, MBP_83–99_ was not immunodominant. Instead, MBP_13–32_, MBP_111–129_, and MBP_146–170_ were immunodominant in these T cells. Higher reactivities to these peptides were seen in MS patients (91).

After promising results in animal models, APLs based on the immunodominant epitope MBP_83–99_ were designed and administered in different phase II clinical trials. In one study, the APL successfully slightly reduced enhancing lesions at a certain dosage and skewed the T-cell response toward a Th2 phenotype, so that the trial had to be stopped because of the occurrence of hypersensitivity reactions (183). However, in another study, the APL induced cross-recognition of MBP_83–99_ and the APL by T cells in some patients, leading to exacerbation (84). One of the patients included in the latter study suffered two relapses under APL-treatment. Interestingly, before the first relapse, the number of T cells specific for MBP_83–99_ increased fivefold to decrease again after remission. In the second relapse, T cells specific for PLP_190–209_ were found expanded (80). This finding shows that myelin reactivity can correlate with disease progression and – besides other pieces of evidence – it also supports the notion of an immune pathogenesis of MS. In other studies, it could also be shown that T-cell reactivities against myelin components can correlate with disease progression, measured by using a disability score (174) or by assessment in MRI (176).

As MBP is an important candidate autoantigen in MS, its relationship with the disease-associated haplotype DR15 is of particular interest. Both HLA-DRB1*1501 and HLA-DRB5*0101 contained in this haplotype can serve as restriction elements for MBP-reactive T cells (177). The immunodominant peptide MBP_85–105_, for example, seems to be able to be presented by both DRB1*1501 and DRB5*0101 (184, 185) and the complex DR2:MBP_85–99_ could be detected in tissue lesions (182).

A recent study showed that MBP is degraded by the 26S proteasome without being ubiquitinated beforehand (186). Whether this discovery can be put in context with the pathogenesis of the disease remains to be resolved, but this shows very well how different aspects of antigen presentation might be involved in the emergence of autoimmune disease.

### Proteolipid protein

Proteolipid protein is the most abundant myelin protein. There are two isoforms: full-length PLP, which is nearly exclusively expressed in the CNS, and DM20, a splice variant of PLP missing a loop of 25 amino acids, which is expressed in various peripheral organs like the thymus and lymph nodes (35). Only DM20 plays a role in negative selection in the thymus (27). The PLP loop contains the epitope PLP_139–151_, which seems predisposed to be a target of autoreactive T cells which can escape central tolerance induction in the thymus. However, enhanced reactivities to this epitope could only be detected in some studies (91), but not in others (181, 187).

T-cell responses to PLP seem to be heterogenous (188). Different epitopes have been identified as immunodominant in different studies (24, 180, 181, 187, 189, 190).

Some studies found higher proliferative responses (191) or higher precursor frequencies (23) of PLP-specific T-cell lines in MS patients. Higher reactivities to some PLP epitopes were detected in some studies in MS patients compared to controls (180, 189). However, other studies could not detect any differences in reactivities against their epitopes detected as immunodominant between patients and controls (188, 190).

### Myelin oligodendrocyte glycoprotein

Myelin oligodendrocyte glycoprotein is a minor component of the myelin sheath. It could not be detected in the human thymus and might therefore evade presentation in central tolerance induction, supporting its possible role as an autoantigen (35). Many different studies have so far assessed T- and B-cell responses to this antigen.

Some studies have detected higher numbers of IFN-γ-secreting cells (192, 193), higher numbers of T cells reactive with DRB1*0401/MOG_97–109_-tetramer (194), higher proliferative responses of peripheral blood lymphocytes (PBL) (195), or higher proliferative responses of T cells to certain MOG epitopes (196) in MS patients than in healthy controls.

Interestingly, there are also data that MOG could play a role in humoral autoimmunity in MS. MOG-specific autoantibodies have been discovered in acute lesions of MS patients (133, 134). Some studies also found higher levels of MOG-specific antibodies or higher antibody responses to certain MOG epitopes in the serum of MS patients than in control sera (197–202) or in certain clinical subgroups of MS patients (203). However, other studies did not observe any differences between the sera of MS patients and healthy controls or patients with other neurological diseases, or did not detect any MOG-antibodies at all (134, 204, 205). The antibodies were shown to be actually demyelinating in an EAE experiment (200).

Data about a possible association of anti-MOG-antibodies (and anti-MBP-antibodies) with a progression from CIS to definite MS remain controversial (202, 206–210), rendering the use of these antibodies as biomarkers difficult.

There are interindividual differences concerning the specific MOG epitopes causing an antibody response (198, 211) and T-cell reactivities (212), which might also depend on the individual HLA type (213).

Important new data also indicate that MOG can interact with DC-specific intercellular adhesion molecule 3-grabbing non-integrin (DC-SIGN), which can induce IL-10 secretion and suppress T-cell proliferation. This interaction depends on the correct glycosylation state of MOG, which can be altered during inflammation (214). These findings could indicate that altered glycosylation of MOG can potentially result in disruption of tolerance and induction of anti-MOG-specific T- and B-cell responses.

### Other and novel autoantigens in MS

Besides the above described antigens MBP, MOG, and PLP, myelin-associated antigen (MAG), myelin-associated oligodendrocyte basic protein (MOBP), and 2′,3′-cyclic-nucleotide 3′-phosphodiesterase (CNPase) have been shown to evoke T- or/and B-cell responses in patients with MS. Apart from these myelin components, other antigens like S100β protein or transaldolase H are discussed as autoantigens in MS (56). While a-B-crystallin was first thought to be an autoantigen in MS (215), more recent data indicate that it serves as a chaperone and does not fulfill the criteria of an autoantigen (216). In another study, 300 peptides presented on MHC class I and MHC class II molecules in the CNS of MS patients were eluted and identified. Among them, widely investigated proteins like MBP were found, but also non-myelin proteins could be detected (217). Table 2 summarizes the autoantigens discussed in MS. For a comparison with other autoimmune diseases, Tables 3 and 4 summarize some important candidate autoantigens in MG, NMO, T1D, RA, and AE.

**Table 2:** **Autoantigens in MS**.

Autoantigen	Remarks	Reference
MBP	T-cell responses and autoantibodies	(91, 175, 311)
MOG	T-cell responses and autoantibodies	(193, 202)
PLP	T-cell responses and autoantibodies	(192, 311)
MAG	T-cell responses and autoantibodies	(312)
MOBP	T-cell responses and autoantibodies	(311, 313)
CNPase	T-cell responses and autoantibodies	(311, 314)
S100β	T-cell responses	(315)
Transaldolase	T-cell responses and autoantibodies	(316)

*For overview of the autoantigens in MS discussed in this review, the respective antigens and the reactions they can evoke in MS patients are listed*.

**Table 3:** **Autoantigens in other (peripheral) autoimmune diseases**.

Autoantigen	Remarks	Reference
**Myasthenia gravis**		
nAChR	Antibodies in most MG patients	(223)
MuSK	Antibodies in “seronegative” MG patients	(222)
LRP4	Antibodies in “seronegative” MG patients	(224, 317)
**Diabetes mellitus type 1**		
Insulin	Antibodies already in prediabetics T-cell reactivities to different epitopes	(253, 254)
IA-2	Antibodies in 50% of diabetics T-cell responses in context of HLA-DR4	(257, 259)
GAD-65	Antibodies in >80% of diabetics Elevated T-cell responses	(256, 260–262)
ZnT8	Antibodies in 60–80% of diabetics at onset of disease Elevated T-cell responses	(258, 263)
IGRP	Elevated T-cell responses	(264)
Chromogranin A	Elevated T-cell responses	(265)
**Rheumatoid arthritis**		
Fc-part of immunoglobulins	Antibodies in >80% of RA patients (rheumatoid factor)	(277)
Citrullinated antigens	Antibodies before and during disease course Specific B cells in synovial fluid	(281, 282)
Carbamylated antigens	Antibodies in 45% of RA patients	(286)
Collagen	Antibodies to post-translationally modified forms Antibodies to denatured forms	(287, 288)
65-kDa heat-shock protein	Antibodies in RA patients	(279)
Cartilage glycoprotein-39	T-cell responses in RA patients	(275)
Aggrecan G1	T-cell responses in RA patients	(276)

*Important (candidate) autoantigens of MG, T1D, and RA are shown. It can be comprehended that in T1D and RA, there are several candidate autoantigens evoking B- and/or T-cell responses, whereas in MG, the target antigens are already more clear*.

**Table 4:** **Autoantigens in NMO and autoimmune encephalitides as examples of other CNS autoimmune diseases**.

Autoantigen	Remarks	Reference
**Neuromyelitis optica**		
AQP-4	Antibodies in 73% of NMO patients	(229)
MOG	Antibodies in 7% of NMO-spectrum disorder patients	(238)
**Autoimmune encephalitides to membrane antigens**		
NMDA-receptor	Antibodies in patients with limbic encephalitis, psychotic behavior	(289, 291)
AMPA-receptor	Antibodies in patients with limbic encephalitis	(289)
GABA_A_-receptor	Antibodies in patients with anti-GABA-A receptor encephalitis	(318)
GABA_B_-receptor	Antibodies in patients with limbic encephalitis	(289)
Gly-receptor	Antibodies in patients with limbic encephalitis, Stiff person syndrome	(289)
DPPX	Antibodies in patients with anti-DPPX-associated encephalitis	(319)
GluR5	Antibodies in patients with anti-GluR5 encephalitis	(320)
VGKC-complex	Antibodies in patients with limbic encephalitis, faciobrachial dystonic seizures, Morvan’s syndrome, neuromyotonia	(289)
**Autoimmune encephalitides to intracellular antigens**		
Hu	T cells and antibodies in patients with anti-Hu encephalitis	(321, 322)
Jo (323, 324), Ri (325), Ma1 (326), Ma2 (327), Zic4 (328), GAD-65 (329), CRMP5 (330), and amphiphysin (331) as target of autoantibodies in patients with the respective encephalitis forms		

## Autoantigens in Other Autoimmune Diseases

### Myasthenia gravis

Myasthenia gravis is a neurological disease characterized by muscle weakness which worsens by exertion and improves by rest (218).

Some decades ago, antibodies directed against nicotine acetylcholine receptors (nAChR) were detected in most MG patients (219, 220), which could also be shown to be pathogenic by a transfer of sera to mice (221). A considerable part of the 10–20% of MG patients who had long been assessed as seronegative for nAChR-autoantibodies could be shown to possess autoantibodies against the receptor tyrosine kinase MuSK (222) or still possess low-affinity antibodies against the nAChR by employing a more sensitive assay (223). More recently, also low-density lipoprotein receptor-related protein (Lrp4) was identified to be a target of autoantibodies in a small proportion of seronegative MG patients (224). These findings point toward B cells playing the central role in the pathogenesis of MG. But also nAChR-specific T lymphocytes have been discovered (22, 225), consistent with necessary T cell help and activation for an autoantibody production by B cells. Regulatory T cells isolated from thymi of MG patients showed a reduced suppressive capability (226).

Even though B cells and their antibodies seem to play a crucial role for the disease, its exact pathogenesis could not be revealed yet. Complement activation by autoantibodies and crosslinking nAChR leading to their degradation were suggested to be underlying pathogenic mechanisms of the nAChR-autoantibodies (218). But still, the emergence of an autoimmune disease always requires an interplay of different immune cell types and T cells are probable to play an important role. Like in MS, as discussed above, it could also be shown that B cells can serve as APCs in MG (227).

Compared to MS, the autoimmune origin of the disease is evidenced very clearly and the nAChR is well characterized as the target structure of the autoimmune response. But still, the exact etiology of the disease remains to be clarified.

### Neuromyelitis optica

Neuromyelitis optica is a demyelinating disease of the CNS characterized by optic neuritis and longitudinally extensive transverse myelitis (228). Originally, the disease was not differentiated from MS, but important differences between NMO and MS will be pointed out in the following. Autoantibodies directed against aquaporin-4 (AQP-4)-channels were discovered in 73% of sera of NMO patients but not in sera of MS patients (229, 230). Anti-AQP-4 antibodies also seem to have some predictive value concerning the clinical course of the disease (231). AQP-4-channels are expressed on astrocytic foot processes at the BBB (230, 232). Pathological findings indicate a complete loss of AQP-4 in NMO lesions, whereas in MS, AQP-4 staining was enhanced in the periplaque white matter of active lesions, and absent in inactive lesions (232). There is evidence that these antibodies might also be pathogenic. They were found to have cytotoxic effects on astrocytes in the presence of complement (233, 234), increase natural killer cell-mediated cytotoxicity (235, 236), complement-mediated involvement of granulocytes (235, 236), and the permeability of the BBB (235). In animal studies, a more severe disease course of EAE displaying features of NMO was observed (236, 237).

There are also NMO patients who are seronegative for AQP-4-antibodies. In 7% of patients with NMO-spectrum disorders (NMOSD), i.e., patients presenting either with isolated transverse myelitis or optic neuritis, MOG-antibodies were discovered (238). Also AQP-4-antibody-negative patients with classical signs of NMO have been described to possess antibodies to MOG (239). However, MOG-antibodies seem to be less specific for NMO. MOG-antibody-positive patients with NMOSD also seem to show a milder clinical course than AQP-4-positive patients (238). Data in animal models of NMO indicate that the T-cell response is not mandatory for the development of NMO if AQP-4 antibodies are present in serum or CSF. The access of the antibodies to the CNS and their binding to astrocytes appears to be most important (240, 241). Consistent with an important role of a loss of B-cell tolerance, regulatory B cells were found decreased in NMO and secreted less IL-10 (97). Nevertheless, T cells seem to be mandatory for the initial emergence of plasma cells which secrete AQP-4-specific antibodies (242, 243).

So even though NMO can sometimes clinically resemble MS, the mechanisms of their pathogeneses seem to differ greatly in regard to the autoimmune target and the emerging immune response.

### Type 1 diabetes

Type 1 diabetes is an autoimmune disease in which the β-cells in the Langerhans islands of the pancreas are destroyed, resulting in a lack of insulin. It is thought that T cells play a more decisive role in β-cell destruction than B cells, but B cells were shown to be relevant by producing autoantibodies against islet cell antigens already in prediabetic phases and by presenting antigen to CD4^+^ and CD8^+^ T cells (244). Concerning CD4^+^ T cells, Th1 and Th17 cells seem to be the most significant T helper cell subsets for T1D. The animal model of T1D is mainly driven by Th1 cells, but there is also evidence of a role of Th1 cells in human T1D (245). More IL-17-secreting T cells were detected in recent-onset T1D and IL-17 seems to promote the inflammatory response to β-cells (246, 247). Th17 cells were also found increased in pancreatic-draining lymph nodes (248). Insulin-specific CD8^+^ T cells could be associated with an autoimmune destruction of transplanted Langerhans islands (249). CD8^+^ T cells were also more frequent in pancreatic-draining lymph nodes than in those of controls (248). Similar to MS, defects in the suppressive capabilities of regulatory T cells have been detected (250). This was also shown for T cells residing in pancreatic-draining lymph nodes (248). On the other hand, also a higher resistance of effector T cells to suppression by regulatory T cells has been reported (251). Possible defects in central and peripheral B-cell tolerance were also reported (141). Epitope spreading also seems to be an important mechanism in the autoimmune response in T1D, intermolecular epitope spreading was observed even before the clinical onset of the disease (252).

Different antigens are discussed as autoantigens in T1D. Among them, insulin seems to play a major role: in some diabetics, antibodies to insulin can be discovered before insulin treatment (253). Most T1D patients were shown to possess CD8^+^ T cells reactive with proinsulin peptides (254).

Islet cell autoantibodies (ICA) were detected to be more frequent in T1D patients and were also suggested to have predictive value (255). Many T1D patients also exhibit antibodies against the enzyme glutamic acid decarboxylase (GAD)-65, which is expressed in neurons and in pancreatic β-cells (256). Subsequently, also autoantibodies against tyrosine phosphatase-like islet-antigen 2 (IA-2) (257) and against the zinc transporter ZnT8 (258) were detected in diabetics.

In addition to insulin, T-cell reactivities against several other antigens were identified: not only autoantibodies, but also T-cell responses against IA-2 and GAD-65 were detected, especially in the context of HLA-DR4 (259–262). Also ZnT8 seems to evoke elevated T-cell responses in diabetics (263). Human PBMCs of patients with recent-onset diabetes also showed elevated IFN-γ secretion when stimulated with islet-specific glucose-6-phosphatase catalytic subunit-related protein (IGRP), supporting a possible role as an autoantigen (264). Chromogranin A was also discovered to be a relevant T-cell antigen in human T1D (265).

On the whole, there are several features MS and T1D have in common. Their animal models seem to be mainly driven by autoreactive Th1 cells, but both animal and human data indicate also an important role of Th17 cells and an impaired function of regulatory populations. Their major genetic risk loci code for MHC molecules and several T- and B-cell antigens have been suggested as putative target structures. Interestingly, T1D has also been reported to display cyclic processes in the ongoing β-cell destruction, characterized by damage by autoreactive T cells, epitope spreading, β-cell proliferation, and suppressive activities by regulatory T cells (266). This could resemble a relapsing-remitting course as found in most MS patients.

### Rheumatoid arthritis

In RA, it is not fully resolved whether T cells or B cells play the more important role in its pathogenesis, for there are important aspects supporting a relevance of both cell types. T- and B-cell clones have been detected in the joints of RA patients, partly even the same clones were found in different affected joints (267, 268). Like in MS and T1D, a pathogenic role of Th1 and Th17 cells has been suggested. Th1 cells were encountered much more frequently than Th17 cells in the joint of RA patients with established disease (269). On the other hand, increased numbers of Th17 cells and higher IL-17 levels were found in peripheral blood and synovial fluid of patients with untreated early RA (270). Part of their pathogenicity might be due to the effect of IL-17, which among other cytokines leads to an increased production of TNF-α (271), which plays a pivotal role in the pathogenesis of RA. Th17 memory cells can also induce secretion of proinflammatory cytokines by synovial fibroblasts (272). Regulatory T cells in RA have been found less able to suppress the production of proinflammatory cytokines like TNFα in acute phases of the disease (273). B cells do not only secrete potentially pathogenic antibodies, but also their function in cellular immunity plays a role in RA, as T cells were shown to be activated by B cells in synovitis (267). Defects in both peripheral and central B-cell tolerance were observed (141). The efficacy of a treatment with rituximab confirms a pathogenic role of B cells in the disease (274).

Like in MS and T1D, several different antigens are discussed as autoantigens in RA; some of the possible targets in RA will be discussed here.

Examples for putative T-cell targets in RA include the human cartilage glycoprotein-39 (275) and aggrecan G1 (276). Some autoantibodies target the Fc-part of immunoglobulins (277). These antibodies are also referred to as the “rheumatoid factor.” The presence of the rheumatoid factor is associated with a more severe disease course (278). Elevated autoantibodies against 65-kDa heat-shock protein have also been detected in RA patients compared to controls (279). An interesting aspect of the autoimmune response in RA is that it also seems to be directed against post-translational modifications of antigens like citrullination or carbamylation (280). Antibodies against different citrullinated antigens are found significantly more often in RA patients compared to controls and are elevated already before the onset of disease (281). A considerable proportion of B cells found in synovial fluid have been observed to secrete antibodies directed against citrullinated antigens (282). Epitope spreading of the antibody response to different citrullinated peptides has been observed even before the clinical onset of RA (283). Data from animal models indicate that antibodies directed against citrullinated antigens are pathogenic and worsen arthritis (284). Human data indicate that anti-cyclic citrullinated antibodies can lead to complement activation (285). Independently from APCA, antibodies directed against carbamylated antigens have been discovered in about 45% of RA patients (286). In early stages of RA, autoantibodies against post-translationally modified collagen have recently been detected in over 90% or RA patients (287). During the course of the disease, also higher antibody titers to denaturated collagen were found in RA patients than in controls (288).

Like T1D and MS, also RA shows an HLA-association, an important role of Th1 and Th17 cells and possible defects in regulatory mechanisms. In contrast to MS and T1D, autoimmune responses to post-translational modifications have been studied more extensively in RA.

### Autoimmune encephalitides

In the last years, several antibody-mediated AE were discovered with immune responses to membrane-bound proteins, which respond well to immunotherapies like plasmapheresis. Symptoms range from psychiatric symptoms, such as personality and behavioral change, agitation and paranoia, to epilepsy, amnesia, and many others. Antibodies against different neuronal antigens were found in different disorders, with most autoantigens directed against certain ion channel proteins or receptors involved in brain signaling. For example, antibodies against *N*-methyl-d-aspartate (NMDA) receptors, alpha-amino-3-hydroxy-5-methyl-4-isoxazoleprionic acid (AMPA)-receptors, gamma-aminobutyric acid (GABA) type A or B receptors, glycine receptors (GlyR), dipeptidyl-peptidase-like-protein 6 (DPPX), metabotrophic glutamate receptor 5 (GluR5), or voltage-gated potassium channels were detected in patients with different forms of autoimmune encephalitis (289).

As an example, only anti-NMDA-receptor encephalitis is briefly discussed here, as there is evidence of a direct pathogenic relevance of the autoantibodies by leading to receptor internalization (290). The occurring autoantibodies were detected to be directed against heteromers of the NMDA-receptor containing the subunits NR2B or NR2A (291). A pathological study barely found markers of cell cytotoxicity, but large deposits of IgG, pointing toward a functionally primary causative role of antibodies produced by B cells instead of cellular immunity (292). Anti-NMDA-receptor encephalitis as well as other types of autoimmune encephalitis can be of paraneoplastic origin. Associations of anti-NMDA-receptor encephalitis with ovarian teratoma have been described (291). The role of T cells in these encephalitis forms is not well established yet. Possibly they are instrumental for disease initiation by helping B cells to secrete pathogenic antibodies.

There are many intracellularly located target antigens that can cause autoimmune encephalitis. Many of these are induced by paraneoplastic mechanisms, but there are also pure autoimmune conditions. Most probably, T cells and especially CD8^+^ T cells are of paramount importance by leading to tissue damage (293, 294). For diagnoses, antibodies against such antigens are pivotal. Antigens that have been described as target antigens and to which also antibodies are found in patients are Hu, Yo, Ri, Ma1, Ma2, Zic4, GAD-65, CRMP5, and amphiphysin (Table 4).

## Ways to the Autoantigens

Principally, possible autoantigens can be determined by testing the immune reaction against antigens that can be assumed to be targeted in an autoimmune disease because of certain characteristics of the disease (e.g., myelin antigens in the demyelinating disease MS). As described above, there has already been much research investigating the role of myelin antigens in MS.

Another way to determine possible autoantigens would be first to identify possible candidate autoantigens of the disease, e.g., by elution of peptides presented on MHC molecules of APCs in affected individuals or by screening sera of patients for autoantibodies, and afterwards test the actual functional role of the findings. The approach to elute peptides presented on APCs in autoimmunity has been successfully applied in research on different autoimmune diseases like RA or T1D (173, 259, 260, 295), but also in MS (217). The eluted peptides from the CNS of MS patients might have a high potential as autoantigens, since antigen presentation in the healthy CNS is low. In active MS lesions, however, MHC molecules have been observed to be upregulated (82).

## Conclusion

In all autoimmune diseases, the importance of the role of peptide–MHC–TCR interactions cannot be underestimated. Antigen presentation is in the center of every autoimmune disease, influencing both tolerance induction in the thymus and self-antigen recognition and immune cell activation in the periphery. This is underlined by the fact that the genetic risk factors of many autoimmune diseases are conferred by genes involved in antigen presentation. The onset of several physiological immune mechanisms like epitope spreading obscures the search for the primary underlying target of the ongoing immune attack. This underlines the great importance of studying immune reactions in recently diagnosed patients or even before diagnosis for eventually being able to determine the primary event in MS and other diseases of a putative autoimmune origin. On the other hand, for more efficient therapy development, the processes which set in later during the course of an autoimmune disease must be taken into account as well. In the discussed autoimmune diseases, the emergence of neo-antigens by tissue destruction and epitope spreading play a role. To give only one example, this might be a reason for the failure of therapeutic trials with peptides based only on MBP in MS treatment. Recently, evidence was adduced that the native conformation of an antigen is of importance for inducing an immune response (296). This is of great relevance for studies investigating possible autoantigens, since antigens are not always employed in their native conformation; which further complicates the search for the principal autoantigen(s).

It is striking that many susceptibility genes conferring risk for different autoimmune diseases code for HLA genes, further emphasizing the extreme relevance of antigen presentation for autoimmune diseases. A comparison of MS with other autoimmune diseases is interesting also in other respects: the exact pathogenesis has not been identified yet in either autoimmune disease. The determination of the (primary) autoantigens is a crucial step in understanding the pathogenesis of a disease. But even when target antigens have been identified like in MG or NMO, the pathogenic mechanism can still remain unclear. Neither has it been resolved which cell types play the dominant role in the induction of an autoimmune disease. The concept of MS being a “T-cell-mediated” autoimmune disease and MG being a “B-cell-mediated” autoimmune disease must be reevaluated. Data indicate that autoimmune diseases emerge from a complex interplay of many different factors of the immune system.

Comparisons of MS with RA or T1D show that there are more diseases whose target antigen(s) have not been fully identified. Until an unequivocal target of the immune attack has been identified in MS and other diseases, there will also remain the question if the causative factors might even be heterogenic.

At all events, a detailed understanding of the etiology of the discussed autoimmune diseases is an enormous challenge, but will remain a prerequisite for the development of new, specific, and more efficient treatment options.

## Author Contributions

CR and RW outlined the subject of the review, searched for, analyzed, and interpreted the literature and wrote the manuscript. CR and RW agree to be accountable for all aspects of the work.

## Conflict of Interest Statement

The authors declare that the research was conducted in the absence of any commercial or financial relationships that could be construed as a potential conflict of interest.
